# Editorial: Regulation of the phenotype and function of human macrophages and dendritic cells by exogenous immunomodulators

**DOI:** 10.3389/fimmu.2023.1353765

**Published:** 2023-12-19

**Authors:** Samer Bazzi, Georges M. Bahr, Nadia Lampiasi

**Affiliations:** ^1^ Department of Biomedical Sciences, Faculty of Medicine and Medical Sciences, University of Balamand, Al Koura, Lebanon; ^2^ Istituto per la Ricerca e l’Innovazione Biomedica, Consiglio Nazionale delle Ricerche, Palermo, Italy

**Keywords:** dendritic cell, macrophage, immunomodulation, anti-inflammatory, hypoxia, toll-like receptor

Macrophages (Mφs) and dendritic cells (DCs) are vital cellular components of the innate immune system whereby they play a central role in tailoring immune responses during states of homeostasis or disease. Mφ and DC responses rely on an array of extrinsic factors that are present in the cellular microenvironment. Therefore, manipulating the extracellular environment through the use of exogenous immunomodulators represents a viable and an innovative strategy to tweak Mφ and DC functions towards eliciting desirable immune responses. Accordingly, the effects of multiple categories of exogenous immunomodulators have been previously evaluated on human immune cells and these include, but are not restricted to, heat-killed mycobacteria (1, 2), phytochemicals (3), biomaterials (4) and toll-like receptor (TLR) agonists (5). In the current Research Topic, six original research articles tackle the aspect of employing previously unexplored exogenous immunomodulators aimed at regulating numerous Mφ- and DC-related activities.

Mφs are distinctly categorized into two types, M1 and M2, in relation to their polarization state. While M1-Mφs display a pro-inflammatory phenotype and retain potent tumoricidal and microbicidal capabilities, M2-Mφs show an anti-inflammatory phenotype and facilitate tumor growth, metastasis development, tissue remodeling and wound healing (6). Gunalp et al. unveil the ability of tumor necrosis factor-related apoptosis-inducing ligand (TRAIL) to drive the polarization of human monocyte-derived Mφs (MDMs) towards the M1-Mφ pro-inflammatory phenotype via upregulating the expression (both at the mRNA and protein levels) of classical and novel M1-Mφ markers on M0-Mφs (unpolarized), M1-Mφs, and different M2-Mφ subtypes without compromising cellular viability. Further functional analysis of TRAIL-treated M1-Mφs shows that these cells exhibit an augmented cytotoxic effect against the acute myeloid leukemia U937 cell line as compared to untreated M1-Mφs. Moreover, this study finds significant positive associations between TRAIL expression and the expression of M1 markers in the tumor microenvironment of ovarian cancer and sarcoma patients as well as the overall survival of a subcategory of those patients who have abundant Mφs in their tumor microenvironment.

Modulation of Mφ function constitutes an immunotherapeutic approach for preventing or treating inflammatory conditions. The fusion protein rFlaA : Betv1, which comprises the adjuvant, flagellin A from *Listeria monocytogenes*, and the key birch pollen allergen Bet v 1, has been previously reported to dampen allergen-induced Th2 inflammation by regulating the release of pro-inflammatory (IL-1β, IL-6 and TNF-α) and anti-inflammatory (IL-10) cytokines by mouse macrophages (7). In their original research article, Lin et al. identify NLRC4 and NLRP3 inflammasomes as essential mediators and modulators of rFlaA : Betv1-induced release of IL-1 β and other pro-inflammatory cytokines by human THP-1-derived Mφs. The authors also demonstrate that rFlaA : Betv1-induced IL-1β secretion from Mφs is highly dependent on NFκB and SAPK/JNK signaling pathways.

The anti-inflammatory effects of cannabinoids have been documented in several *in vitro* and *in vivo* studies (8). In line with this, Perez-Diego et al. investigate the mechanisms through which the synthetic cannabinoid, WIN55,212-2, induces its anti-inflammatory effects on various human myeloid cells. Interestingly, the authors find that human monocyte-derived DCs (MDDCs), differentiated in the presence of WIN55,212-2, produce a tolerogenic DC type that is characterized by its diminished responses to LPS and by its capacity to prime Tregs. Results also show that WIN55,212-2 perturbs the polarization of human THP-1-derived Mφs and MDMs towards the pro-inflammatory M1-Mφ type via impairing the LPS-induced intracellular metabolic and epigenetic reprogramming of Mφs. This leads to the inhibition of their pro-inflammatory cytokines secretion, pyroptosis and inflammasome activation.

Plasmacytoid dendritic cells (pDCs) are professional antigen-presenting cells (APCs) able of playing an important role in directing the immune response to antigens. TLR-activated pDCs exhibit robust IFN-α production and promote both innate and adaptive immune responses. PDCs respond to viral infections (DNA and RNA) by producing large quantities of IFN-α through the stimulation of TLR7 and TLR9 (9). Mechanistically, transcription of the IFN-α gene results in activating the transcription of pro-inflammatory cytokines (10). In viral infections, type I IFNs are known to play a protective role though there is growing evidence that chronic secretion of IFN-α results in pathological inflammation (11) and autoimmune diseases such as SLE (12). However, it is still unclear which mechanism controls pDCs’ selective cytokine production. One possibility could be related to the localization process of TLR7 and TLR9 agonists in intracellular compartments. Two original research articles shed light on the molecular mechanisms of the bifurcated cytokines responses to TLR7 and TLR9 agonists in pDCs (Wiest et al. and Wiest et al.). EGA (4-bromobenzaldehyde N-(2,6-dimethylphenyl)semicarbazone), an inhibitor of endosomal trafficking, is used in these studies to assess its disruptive effects on TLR7/9 agonist-induced cytokine responses in pDCs from healthy donors and SLE patients. The results herein highlight that EGA can decrease the expression of IFN-α in cells from healthy donors and SLE patients by TRL7 and TLR9 agonists. EGA works by reducing the localization of TRL7 and TLR9 agonists in late endosomes/lysosomal compartments without altering the retention of agonists in early/recycling endosomes. Mechanistically, EGA treatment decreases phosphorylation of IKKα/β, STAT1, and p38, and prolongs degradation of IκBα. The conclusion supported by these studies is that lysosome associated membrane protein-1 positive (LAMP1^+^) compartments (late/lysosomes) are important for the expression of IFNα by pDCs. Therefore, inhibitors of this process, such as EGA, may be beneficial in the future treatment of inflammatory diseases associated with type 1 IFNs.

Previous studies have pointed out to the profound negative impact of the hypoxic tumor microenvironment on various functional features of tumor-resident DCs (13). Bhatt et al. investigate whether O_2_-cryogels, an O_2_-releasing biomaterial, can prevent hypoxia-induced suppression of human DC functions. Study results indicate that exposure of human MDDCs to O_2_-cryogels counterbalances hypoxia-induced inhibition of antigen uptake, maturation state and migratory activity in DCs. Moreover, O_2_-cryogels possess immunomodulatory properties that preserve DC’s capacity to efficiently prime naive T cells under hypoxic conditions and, consequently, induce their activation and proliferation.

In summary, this Research Topic provides a deep insight into the effects induced by novel exogenous immunomodulators on human Mφs and DCs and subsequently shaping immune responses. The use of such types of exogenous immunomodulators holds a promising therapeutic strategy for treating inflammatory disorders and cancer.

## Author contributions

SB: Writing – original draft, Writing – review & editing. GB: Writing – original draft, Writing – review & editing. NL: Writing – original draft, Writing – review & editing.
